# The Protective Effect of *Bcl-xl* Overexpression against Oxidative Stress-Induced Vascular Endothelial Cell Injury and the Role of the Akt/eNOS Pathway

**DOI:** 10.3390/ijms141122149

**Published:** 2013-11-08

**Authors:** Leng Ni, Tianjia Li, Bao Liu, Xitao Song, Genhuan Yang, Linfang Wang, Shiying Miao, Changwei Liu

**Affiliations:** 1 Department of Vascular Surgery, Peking Union Medical College Hospital, Chinese Academy of Medical Science, Peking Union Medical College, 1# Shuaifuyuan, Dongcheng District, Beijing 100730, China; E-Mails: pumchnileng@gmail.com (L.N.); litianjia2008@163.com (T.L.); dr.liubao@gmail.com (B.L.); sxitao@sina.com (X.S.); lkyanggh1984@163.com (G.Y.); 2 National Laboratory of Medical Molecular Biology, School of Basic Medicine, Peking Union Medical College, Chinese Academy of Medical Sciences, Beijing 100730, China; E-Mails: wang.linfang@imicams.ac.cn (L.W.); miaosywyd@163.com (S.M.)

**Keywords:** vascular endothelial cells, oxidative stress, intimal hyperplasia, gene therapy, apoptosis

## Abstract

Restenosis after intraluminal or open vascular reconstruction remains an important clinical problem. Vascular endothelial cell (EC) injury induced by oxidative stress plays an important role in the development of intimal hyperplasia. In this study, we sought to evaluate the protective effects of *Bcl-xl* overexpression *in vitro* on oxidative stress-induced EC injury and the role of the Akt/endothelial nitric oxide synthase (eNOS) pathway. Human umbilical vein endothelial cells (HUVECs) exposed to hydrogen peroxide (H_2_O_2_, 0.5 mM) were used as the experimental oxidative stress model. The *Bcl-xl* gene was transferred into HUVECs through recombinant adenovirus vector pAdxsi-GFP-*Bcl-xl* before oxidative treatment. Cell apoptosis was evaluated by Annexin V/propidium iodide and Hoechst staining, caspase-7 and PARP cleavage. Cell viability was assessed using the cell counting kit-8 assay, proliferating cell nuclear antigen (PCNA) immunocytochemical detection and the scratching assay. Expressions of Akt, phospho-Akt and eNOS were detected by Western blotting. Our results showed that H_2_O_2_ induced apoptosis and decreased the cell viability of HUVECs. *Bcl-xl* overexpression significantly protected cells from H_2_O_2_-induced cell damage and apoptosis and maintained the cell function. Furthermore, the level of phospho-Akt and eNOS protein expression was significantly elevated when pretreated with *Bcl-xl* gene transferring. These findings suggest that *Bcl-xl* overexpression exerts an anti-apoptotic and protective effect on EC function. The Akt/eNOS signaling pathway is probably involved in these processes.

## 1. Introduction

Peripheral arterial disease (PAD) is a devastating disease with a major effect on limb salvage and the quality of life. Especially among the population with risk factors of atherosclerosis, the incidence of PAD is rather higher than the general population. Guan *et al*. [1] investigated the prevalence of PAD in diabetic patients over 50 years old in China. In total, of 1397 diabetic patients aged 50 years, 272 (19.5%) patients were diagnosed as having PAD. With the progressing of aging, the number of patients with PAD and the number of peripheral vascular interventions performed each year will increase all over the world. These interventions may include angioplasty, endarterectomy or autologous or prosthetic graft bypass. The long-term patency after any intervention is compromised by the development of intimal hyperplasia (IH) and the consequence event of it, such as the limitation of the inflow or outflow or thrombosis in the grafts [2]. Until now, it has been the most difficult problem in clinical practice for vascular surgeons.

Though the mechanism of IH is still unknown, vascular endothelial cells (ECs) injury or dysfunction is the triggering event of all the following pathophysiological processes, such as platelet adhesion, infiltration of inflammatory cells and proliferation of vascular smooth muscle cells (VSMC) [3,4]. Oxidative stress accompanied with surgical intervention is an important reason for ECs apoptosis or injury [2]. Some studies have indicated that apoptotic ECs display noticeable procoagulant activities and become pro-adhesive towards platelets and inflammatory cells [5,6]. Therefore, protection of ECs from oxidative injury and apoptosis should prove to be a beneficial strategy for the treatment of IH. *Bcl-xl*, as a number of the *Bcl-2* family, is thought to play an important role in reducing oxidative stress-induced apoptosis through maintaining mitochondrial homeostasis [6]. Previous studies have shown that anti-apoptotic proteins, such as *Bcl-xl*, can decrease the level of apoptosis after heart transplantation, thus effectively reducing reactive oxygen species (ROS)-induced myocardial reperfusion injury and improving cardiac function [7]. However, less evidence is available about the cytoprotective effect of *Bcl-xl* on vascular ECs under oxidative circumstances. In this study, we aimed to verify if delivering *Bcl-xl*, an anti-apoptotic gene to primary ECs, can ameliorate the injury caused by oxidative stress and sustain the normal function of ECs.

## 2. Results and Discussion

### 2.1. *Bcl-xl* Overexpression Attenuates Oxidative Injury-Induced Apoptosis in HUVECs

**Figure 1:**
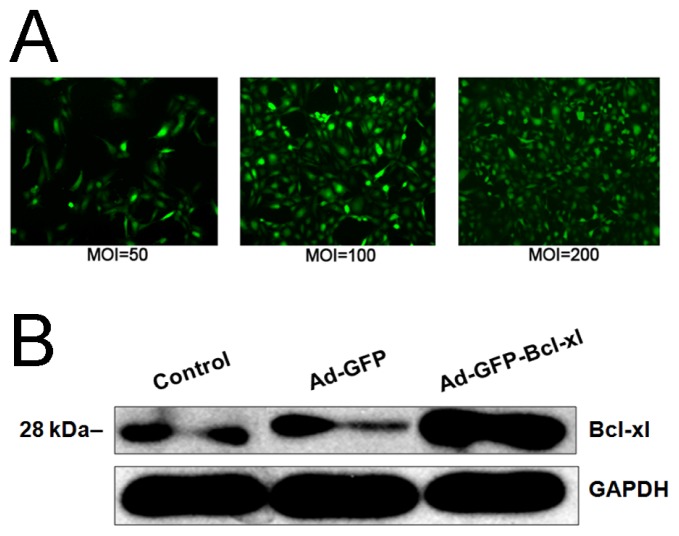
HUVECs infection with recombinant adenovirus (Adv-GFP-*Bcl-xl*) or empty virus (Adv-GFP) and the expression of *Bcl-xl* by Western blotting. (**A**) Observation of HUVECs infected at different multiplicities of infection (MOI) for 48 h by fluorescence microscopy (×40). Green fluorescence represents infected cells; (**B**) *Bcl-xl* expression in HUVECs of different groups by Western blotting.

Human umbilical vein endothelial cells (HUVECs) were infected with pAdxsi-GFP-*Bcl-xl* at various multiplicities of infection (MOI) for 48 h, and their *Bcl-xl* expression was detected by Western blotting. The results showed that high levels of *Bcl-xl* were expressed in pAdxsi-GFP-*Bcl-xl*-infected cells, but not in normal cells or HUVECs infected with empty vector (Figure 1).

**Figure 2:**
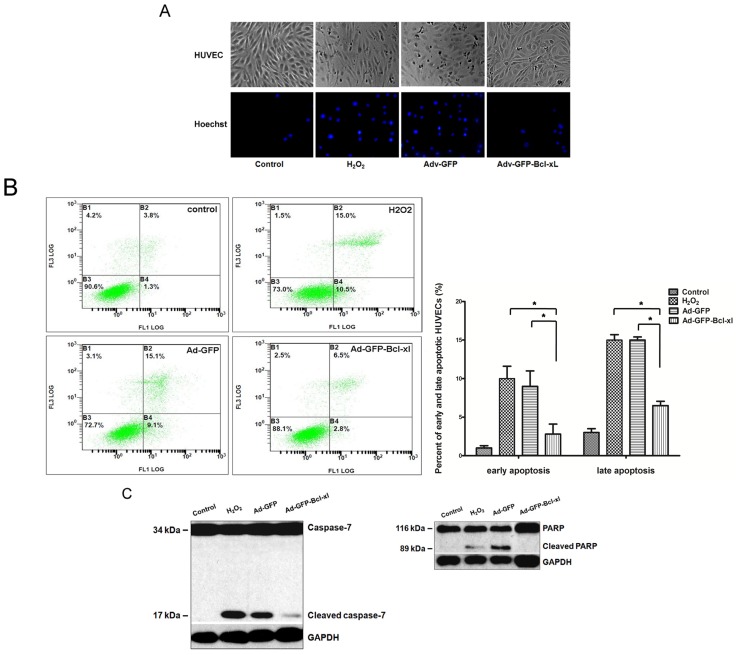
Evaluation of cell apoptosis level in each group. After being infected with Adv-GFP-*Bcl-xl* (or Adv-GFP) of 100 MOI for 48 h, HUVECs were subsequently stimulated with 0.5 mM H_2_O_2_ for 12 h. (**A**) Observation of cell morphological changes under microscope (×40) and nuclear staining with Hoechst 33,258 (×100); (**B**) Analysis of HUVEC apoptosis through Annexin V-fluorescein isothiocyanate/propidium iodide(Annexin V-FITC/PI) flow cytometry. The proportion (%) of the cell number is shown in each quadrant. The proportion of viable cells was shown in the B3 quadrant (FITC−/PI−). Early apoptotic cells are shown in the B4 quadrant (FITC+/PI−), and late apoptotic/necrotic cells are shown in the B2 quadrant (FITC+/PI+); (**C**) Western blot analysis of the apoptotic-related proteins, caspase-7 and PARP, in HUVECs. Control: normal HUVECs; H_2_O_2_: HUVECs exposed to 0.5 mM H_2_O_2_; Adv-GFP: HUVECs pretreated with empty virus and then exposed to 0.5 mM H_2_O_2_; Adv-GFP-*Bcl-xl*: HUVECs pretreated with Adv-GFP-*Bcl-xl* and then exposed to 0.5 mM H_2_O_2_. Data are representative of the means ± the standard error of the mean (SEM) (*n* = 6). Statistical analysis was performed using one-way analysis of variance (ANOVA), followed by Tukey’s multiple comparison test (*p < 0.05).

The normal HUVECs manifest middle-sized, short fusiform cells, which seemed like “cobblestone” under microscopy. After adding H_2_O_2_, cells were deformed and contracted, had increased intracellular granular material and showed a roughened profile (Figure 2A). Additionally, Hoechst 33,258 staining shows nuclear condensation in HUVECs. As shown in Figure 2A, the nuclei of HUVECs in the H_2_O_2_ and Adv-GFP group (H_2_O_2_ 0.5 mM) showed brightly stained condensed chromatin with Hoechst 33,258. In contrast, HUVECs that delivered *Bcl-xl* were lightly stained with Hoechst (rare cells with condensed and brightly stained chromatin), which showed that *Bcl-xl* overexpression inhibited oxidative injury-induced apoptosis.

To determine whether the oxidative injury on HUVECs is due to the induction of cell death, the cell apoptosis rate was measured by Annexin V/propidium iodide(Annexin V/PI) staining. As shown in Figure 2B, the proportion of early apoptotic cells labeled with annexin V was increased from 1.3% ± 0.3% in the control to 10.5% ± 1.6% in the H_2_O_2_ group and 9.1% ± 2.0% in the Adv-GFP group. In contrast, the proportion in the Adv-GFP-*Bcl-xl* group was decreased to 2.8% ± 1.4% (*p* < 0.05 *vs*. the H_2_O_2_ group and the Adv-GFP group). *Bcl-xl* overexpression appeared also to decrease the late apoptosis rate according to the result of PI staining. These results suggested that *Bcl-xl* overexpression could reduce the proportion of cells entering early and late apoptosis to the level of untreated HUVECs.

To further examine cell apoptosis at the molecular level, we examined the apoptosis-related protein, caspase-7, and PARP by Western blotting. The cleavage band of caspase-7 and PARP was observed at 12 h after treatment with H_2_O_2_ (Figure 2C). However, the expression level of cleaved caspase-7 and PARP in the Adv-GFP-*Bcl-xl* group was lower than the H_2_O_2_ and Adv-GFP group. These results suggested that the mitochondria pathway was involved in oxidative injury-induced EC apoptosis, and overexpression of *Bcl-xl* significantly protected HUVECs from apoptosis in oxidative stress models.

### 2.2. Protective Effect of *Bcl-xl* Overexpression on Maintaining the Viability of HUVECs

**Figure 3:**
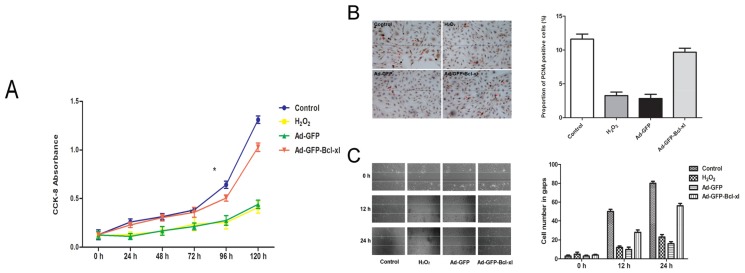
Evaluation of cell viability in each group. After being infected with Adv-GFP-*Bcl-xl* (or Adv-GFP) of 100 MOI for 48 h, HUVECs were subsequently stimulated with 0.5 mM H_2_O_2_ for 12 h. Cell proliferative function is performed by (**A**) The CCK-8 assay and (**B**) Immunocytochemical stain of proliferating cell nuclear antigen (PCNA) in HUVECs (×40). The red arrow represents PCNA positive cells; (**C**) Evaluation of cell migration functions by the scratching test. After being pretreated as described in the Methods section, we scratched the cells by using a pipette tip. Representative pictures were taken at 0 h, 12 h and 24 h. The cell number was counted in the scratched gaps. Control: normal HUVECs; H_2_O_2_: HUVECs exposed with 0.5 mM H_2_O_2_; Adv-GFP: HUVECs pretreated with empty virus and then exposed with 0.5 mM H_2_O_2_; Adv-GFP-*Bcl-xl*: HUVECs pretreated with Adv-GFP-*Bcl-xl* and then exposed with 0.5 mM H_2_O_2_. Results are expressed as the means ± SEM (*n* = 6). Statistical analysis was performed using one-way analysis of variance (ANOVA) followed by Tukey’s multiple comparison test (*p < 0.05).

The result of the growth curve through the Cell Counting Kit-8 (CCK-8) assay (Figure 3A) showed that oxidative stress significantly decreased cell viability compared with untreated cells. However, the cell proliferation speed of the Adv-GFP-*Bcl-xl* group was higher than the H_2_O_2_ group and the Adv-GFP group, especially from the fourth day of exposer to H_2_O_2_. The growth curve of the Adv-GFP-*Bcl-xl* group is almost consistent with the control. In immunocytochemical detection (Figure 3B), the proportion of proliferating cell nuclear antigen (PCNA) positive cells in Adv-GFP-*Bcl-xl* group was also higher than the other two groups. These results suggested that *Bcl-xl* overexpression could accelerate the regeneration of ECs under the circumstances of oxidative stress.

The results of the cell scratching assay (Figure 3C) revealed that the degrees of wound healing in normal cells were about 50% after 12 h, and after 24 h, the wound healed completely. In oxidative conditions, the speed of wound healing slowed down significantly. The degree of healing was still less than 50% after 24 h. The wound healing of the Adv-GFP-*Bcl-xl* group was significantly accelerated compared to the H_2_O_2_ group and the Adv-GFP group, almost consistent with the control.

### 2.3. Involvement of the Akt/eNOS Pathway in *Bcl-xl* gene Transfected H_2_O_2_-Stimulated HUVECs

**Figure 4:**
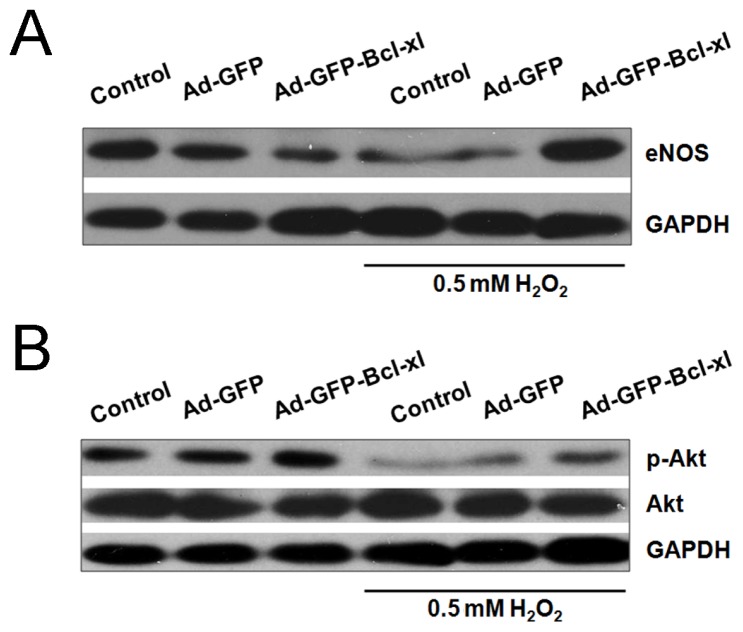
The effects of *Bcl-xl* overexpression on Akt phosphorylation and eNOS protein levels in H_2_O_2_-stimulated HUVECs. After being infected with Adv-GFP-*Bcl-xl* (or Adv-GFP) of 100 MOI for 48 h, HUVECs were subsequently stimulated with 0.5 mM H_2_O_2_ for 12 h. Equal amounts of protein were resolved by SDS-PAGE followed by Western blot analysis with antibodies against each specific protein. (**A**) Western blotting for the measurement of eNOS protein levels in HUVECs; (**B**) Western blotting for the measurement of Akt and p-Akt protein levels.

Western blotting demonstrated that the protein level of endothelial nitric oxide synthase (eNOS) in Adv-GFP-Bcl-xl was higher than the H_2_O_2_ group and the Adv-GFP group, though it decreased in the control (Figure 4A). Phosphorylation levels of Akt were significantly increased in *Bcl-xl* overexpressed HUVECs, whereas in non-infected H_2_O_2_-stimulated cells, the level of phospho-Akt was lower than the control (Figure 4B). These results suggest that *Bcl-xl* overexpression blocked the eNOS and phospho-Akt inhibition in response to H_2_O_2_.

### 2.4. Discussion

In the present study, we showed that adenovirus-mediated gene transfer of *Bcl-xl* in HUVECs not only attenuates ECs injury induced by oxidative stress, but also maintains its normal physiological functions, such as proliferation, migration and synthesis, probably through the Akt/eNOS signaling pathway. These results might provide an effective therapeutic adjunct to facilitate the transfer of experimental treatments for vascular injury to the clinic.

Restenosis caused by IH after surgical procedures, such as bypass or stenting, severely limits the overall efficacy of these interventions and can occur in up to 80% of patients [8]. Although the pathophysiology of IH is complex, ECs injury and loss of integrity are often regarded as the triggering events for the development of IH. ECs act as an important biological barrier, protecting against platelet and monocyte adhesion and inhibiting the proliferation of vascular smooth muscle cells (VSMCs) [9]. The apoptosis of ECs may destroy the vascular barrier and expose VSMCs to the blood stream directly, permitting the adhesion and activation of platelet and inflammatory cells [10–12]. Activated inflammatory cells generate excessive ROS in the vascular wall, which includes H_2_O_2_ and superoxide anions. On one hand, these ROS lead to detrimental consequences, including cell apoptosis and injury [13]; on the other hand, ROS induce the production of many cytokines and inflammatory factors [14,15]. For example, the production of interleukin-8, which is a potent neutrophil chemoattractant and activator, is upregulated by ROS [16]. Platelet activating factor (PAF), monocyte chemoattractant protein-1 (MCP-1) and monocyte colony-stimulating factor (MCSF) are also induced by ROS [17,18]. These chemoattractants may gather more neutrophils and monocytes at injury sites. This vicious cycling leads to the production of large amounts of ROS, which can continually accelerate oxidative damage to ECs and delay the re-endothelization of injured vascular or prosthetic graft and, finally, lead to IH.

Ischemia-reperfusion (I/R) injury and inflammation followed by surgical procedures and many atherosclerosis risk factors, such as diabetes, hypertension, hyperlipidemia and smoking, can generate heightened ROS [18–23]. Thus, the inhibition of ROS formation and the scavenging of ROS, protecting ECs from apoptosis elicited by oxidative stress, might be the potential ways to promote re-endothelization and to prevent IH. Once endothelial cell regeneration is complete, the stimulus for platelet adherence and leukocyte chemotaxis is removed, and the cascade of cytokines and growth factor release leading to neointimal hyperplasia is abolished [24]. Convincing data has been accumulated in the treatment of oxidative stress-induced cell injury using antioxidant drugs [25–27]. For example, *N*-acetylcysteine (NAC) and probucol therapy are associated with a decrease in oxidative stress and improved graft endothelialization in hypercholesterolemic rabbits [2]. Propofol significantly protected cells from H_2_O_2_-induced cell damage and apoptosis, decreased caspase-3 activity and significantly increased eNOS expression compared to the control and H_2_O_2_-stimulated cells [28]. However, the anti-oxidative effects under systematic application of antioxidants can hardly be obtained, because of the lower drug concentration in the local region of the vascular wall. Local drug delivery can result in drug concentrations in vascular tissues that are 400–1000 higher than that achieved following systemic administration of the same compound [29]. With the development of gene therapy, locally transferring target genes into the vascular wall is gradually becoming a common method for preventing restenosis.

Previous studies have demonstrated that the *Bcl-2* family is close relative to apoptosis in vascular injury. For example, Shibata *et al*. [30] observed that balloon injury resulted in a small increase in *Bax* expression and a significant increase in *Bcl-xl* expression estimated 14 days after balloon injury. However, less information is available on the study of the anti-apoptosis effects of *Bcl-xl* on vascular ECs induced by oxidative stress. Moreover, the cytoprotective effect of *Bcl-xl* on ECs has not been explored. In this study, we first investigated the anti-apoptotic effects of *Bcl-xl* on ECs *in vitro* and observed if *Bcl-xl* overexpression favors improving endothelial regeneration, accelerating the recovery of endothelium-dependent biological functions.

H_2_O_2_ is known to be one of the common forms of ROS and can easily penetrate the plasma membrane and affect neighboring cells [31]. Therefore, in this study, H_2_O_2_ was used in the design of an oxidative stress model in endothelial cells. In order to verify if *Bcl-xl* overexpression can inhibit HUVEC apoptosis induced by H_2_O_2_, we evaluated the level of apoptosis from two aspects, which are cell morphology and apoptotic-related proteins. Firstly, we observed that ECs exposed to H_2_O_2_ were deformed and contracted, had increased intracellular granular material and showed a roughened profile. These phenomena can be explained by the previous study, which showed that oxidative stress can result in the alteration of cell shape, the recombination of actin filaments and the formation of intercellular gaps [32]. In addition, apoptotic cells manifest nuclear condensation, which can be assessed by Hoechst staining. HUVECs in oxidative groups showed brightly stained condensed chromatin with Hoechst stain. In contrast, the proportion of nuclei labeled with Hoechst in *Bcl-xl* overexpressed HUVECs was lower than other groups. *Bcl-xl* overexpression can decrease the rate of the early and late cell apoptosis rate through Annexin V/PI staining and measuring by a flow cytometer. Furthermore, we evaluated the level of apoptotic-related proteins. Caspases, a family of specific cysteine proteases, are critical mediators of apoptosis [33]. Oxidative injury can activate the caspase family proteins through the mitochondria pathway [34]. In our study, we evaluated the activated level of caspase-7 and PARP through measuring the expression of cleaved protein by western blotting. Both cleaved caspase-7 and cleaved PARP in HUVECs exposed to H_2_O_2_ are more highly expressed than control cells. On the other hand, the HUVECs infected with Adv-GFP-*Bcl-xl* had a significantly lower expression of cleaved apoptotic-related proteins.

Some evidence was manifested that ECs layer regeneration needs only two to four weeks after injury [35], but the regenerating endothelium experiences dysfunction in the first three months, characterized by decreased endothelial integrity and vascular protective factors, impaired endothelium-dependent vasodilatation and increased permeability [3]. The impaired cell physical functions can promote the delay of endothelium repair. Therefore, the true meaning of “re-endothelialization” involves not only having intact ECs in the anatomical structure, but more importantly, it involves promoting the functioning of ECs back to normal as soon as possible. In order to identify if transferring the *Bcl-xl* gene can maintain normal ECs functions while inhibiting ECs apoptosis and sustaining endothelial integrity, we assessed the following cell functions.

To assess the viability and proliferative functions, we described the growth curves though the CCK-8 assay and measured the proportion of PCNA positive cells by immunocytochemical detection. Furthermore, we evaluated the immigration function of ECs through the scratching test. We found that, after transferring the *Bcl-xl* gene to ECs, the influence of oxidative injury on ECs function is weakened, not only in the viability and proliferation, but also in the migration function.

NO synthesized by endothelial nitric oxide synthase (eNOS) is capable of inhibiting apoptosis and is regarded as an endothelial cell survival factor [36]. NO has several biochemical activities, including mediating vasodilation, directly scavenging superoxide, attenuating leukocyte adhesion and activation and maintenance ofendothelial integrity [37]. The Akt/eNOS/NO pathway plays a key role in preventing ROS-induced endothelial cell injury [38]. A previous study showed that eNOS knockout mice that underwent external carotid artery ligation displayed an increase in wall thickness and a hyperplastic response of the arterial wall [39]. Perivascular application of NO-releasing self-assembling nanofiber gels is an effective therapy to prevent neointimal hyperplasia after arterial injury [40]. In our study, *Bcl-xl* overexpression can block the eNOS and phospho-Akt inhibition under oxidative circumstances, but cannot affect Akt phosphorylation and eNOS protein expression under normal conditions. These results showed that *Bcl-xl* maintains EC physiological functions, probably by activating the Akt/eNOS signaling pathway.

## 3. Experimental Section

### 3.1. Culturing of HUVECs

HUVECs were isolated from umbilical vein cords of normal pregnancies following a protocol described previously [41]. These cells were cultured on flasks coated with 0.2% gelatin in medium Dulbecco’s Modified Eagle Medium (DMEM) supplemented with 10% fetal bovine serum (Gibco BRL, Gaithersburg, MD, USA) and 15 mg/L of Endothelial Cell Growth Supplement (ECGS) (ScienCell, Carlsbad, CA, USA) in an atmosphere of 5% CO_2_ at 37 °C. The medium was changed every 3–4 days until the cells reached confluence. To maintain a uniform condition, all experiments were carried out between cell passages 4 and 6.

### 3.2. Generation of Recombinant Adenovirus Vectors

The DNA fragment of human *Bcl-xl* was generated by polymerase chain reaction, using template cDNA from HUVECs, using the following primers: 5′-ACCTGCCTGCCTTTGCCTAA-3′ and 5′-AGGGTAGAGTGGATGGTCAGT-3′. Then, the DNA fragment with *Bcl-xl* was connected into vector pGEM-T Easy vector in the condition of DNA ligase, which generated pGEM-T-*Bcl-xl*. The EagI restriction enzyme cutting fragment of pGEM-T-*Bcl-xl* was inserted into the NotI restriction enzyme cutting site of pShuttle-GFP-CMV, which generated pShuttle-GFP-CMV-*Bcl-xl*. The pAdxsi-GFP-*Bcl-xl* cosmid was constructed by inserting the I-CeuI/I-SceI site of pShuttle-GFP-CMV-*Bcl-xl* into the I-CeuI/I-SceI site of the cosmid pAdxsi, which was provided by SinoGenoMax group Research Center Corporation. Empty vector pAdxsi-GFP was used for the control. The recombinant adenovirus was generated by transfection and amplified in human embryonic kidney 293 cells. Viruses were purified by CsCl density gradient centrifugation, and virus titer was measured by TCID50 protocol.

### 3.3. HUVECs Infection with Recombinant Adenovirus

For infection, ~3 × 10^5^ cells were seeded on a 6 cm^2^ Petri dish and treated with selected adenovirus at different multiplicities of infection (MOI) in the range 50 to 200 for 48 h. The culture medium was changed for incubation longer than 36 h. Infection efficiency was determined by flow cytometry. The expression of *Bcl-xl* in HUVECs after infection was assessed by western blotting.

### 3.4. Treatment of HUVECs with Hydrogen Peroxide

The HUVECs were seeded onto gelatin-coated tissue culture plates and incubated with the medium containing 0.5 mM hydrogen peroxide for 12 h and harvested for the following experiments.

### 3.5. Analysis of Apoptosis by Hoechst 33,258 and Annexin V/PI Staining

DNA chromatin morphology was assessed using Hoechst staining. After being exposed to H_2_O_2_, the cells were washed by phosphate buffered saline (PBS) and labelled with 5 μg/mL of Hoechst 33,258 for 10 min, and the cells were examined by fluorescence microscopy. After the oxidative event, the cells were stained for 7 min at 37 °C with 1:100 annexinAnnexin V-FITC (R & D Systems Europe Ltd., Abingdon, UK) in phosphate buffered saline (PBS) solution. After Annexin V-FITC staining, the cells were treated with 20 μg/mL PI (SIGMA, Shanghai, China) for 3 min at 37 °C; the cells was subsequently counted by flow cytometry (Coulter, Hialeah, FL, USA).

### 3.6. Analysis of Protein Levels by Western Blotting

HUVECs were exposed to the various experimental conditions for predetermined periods. Then, the cells were harvested and lysed in RIPARadioimmunoprecipitation assay (RIPA) buffer (Boston Bioproducts, MA, USA) for total protein extraction. The protein concentration of each sample was determined by the Bicinchoninic Acid (BCA) Protein Assay kit (Pierce Biotechnology, Rockford, IL, USA). Equal amounts of protein (10 μg) were subjected to electrophoresis on 10% SDS-polyacrylamide gels and transferred to a polyvinylidene difluoride (PVDF) membrane (Millipore, MA, USA). After being blocked for 1 h in Tris-Buffered Saline Tween-20 (TBST) with 5% nonfat milk, the PVDF membrane was then probed with primary antibodies (Bcl-xl, PARP, eNOS, Akt and phospho-Akt antibodies were from Cell Signaling, 1:1000; caspase-7 antibodies were from MBL, 1:1000) overnight at 4 °C and then with horseradish peroxidase (HRP) conjugated secondary antibodies for 1 h at room temperature. The blots were detected with the enhanced chemiluminescence (ECL) assay kit (Santa Cruz Biotechnology, Inc., Heidelberg, Germany).

### 3.7. Cell Viability Assay

The CCK-8 assay was used to estimate cell viability. HUVECs were seeded on a 96-well plate (2 × 10^4^ cells/well). Thirty-six hours after adenoviral infection, the cells were pretreated with 0.5 mM H_2_O_2_ and cultured at 37 °C for an additional 12 h before the addition of CCK-8 solution (stock solution of 10 mg/mL). At the different time points (0 h, 24 h, 48 h, 72 h, 96 h, 122 h), twenty microliters of the CCK-8 stock solution was then added into each well to attain a total reaction volume of 200 μL. After incubating for 3 h at 37 °C, the plates were gently shaken for 10 min and were read at 450 nm on a scanning multi-well spectrophotometer.

### 3.8. Immunocytochemical Detection

HUVECs with or without adenoviral infection were washed with PBS and fixed with 4% paraformaldehyde in PBS at room temperature (RT) for 15 min. The samples were treated with PBS containing 1% Triton X-100 for 5 min and then blocked with PBS containing 5% bovine serum albumin (BSA) at RT for 1 h. Rabbit anti-human proliferating cell nuclear antigen (PCNA) antibody (1:20, Santa Cruz Biotechnology, Paso Robles, CA, USA) was added and incubated for 1 h. After rinsing with PBS, the cells were incubated in a 1:500 dilution of biotinylated secondary antibody (Vector Laboratories, Burlingame, CA, USA) at 37 °C for 1 h. Following a washing with PBS, the cells were treated further with an avidin-biotin conjugated to horseradish peroxidase, developed with 3,3′-Diaminobenzidine (DAB) (Vector Laboratories, Burlingame, CA, USA) and counterstained with hematoxylin.

### 3.9. Scratching Assay

The cell migration function was measured by wound healing assay. Equal numbers of cells (5 × 10^5^) were plated in 6-well plates. After being pretreated with 0.5 mM H_2_O_2_, the monolayer of the cells were wounded by manual scratching with a pipette tip, then photographed in phase contrast with a Nikon microscope (0 h point) and placed into complete growth medium. Matching wound regions were photographed after 0, 12 and 24 h.

### 3.10. Statistical Analysis

All data from three separate experiments are given as the mean ± SEM and analyzed using one-way ANOVA for comparisons of group means. For all analyses, differences were considered significant at *p* < 0.05. All statistical analyses were conducted using the Statistical Program for Social Sciences 13.0 software program (SPSS Inc., Chicago, IL, USA, 2004).

## 4. Conclusions

In summary, the present study proved that adenovirus-mediated gene transfer of *Bcl-xl* in HUVECs could attenuate oxidative-induced apoptosis and maintain ECs functions. The activation of the Akt/eNOS/NO pathway might hold potential for vasoprotection against oxidative damage. These findings may provide additional insight to vasoprotection and support of this novel gene therapy approach in the treatment of intimal hyperplasia and restenosis and needs to be tested *in vivo* and in clinical trials.
